# Interpreting the inner world of ADHD children: psychoanalytic perspectives

**DOI:** 10.1080/17482631.2017.1298269

**Published:** 2017-05-22

**Authors:** Björn Salomonsson

**Affiliations:** ^a^ Department of Women’s and Children’s Health, Karolinska Institute, Stockholm, Sweden

**Keywords:** Child psychotherapy, child psychoanalysis, qualitative method, hermeneutics, and neuropsychiatry

## Abstract

ADHD is increasingly seen as associated with cerebral dysfunction and caused by it. This development is concomitant with an emphasis on medication, behavioural treatments, and parent training programmes. In contrast, psychoanalytic therapy has receded into the background and is often viewed as inefficient or even noxious. This paper argues that such views are based on a misunderstanding of the scope of psychotherapy. Though much more systematic research is needed to establish its efficacy, it can inform on the ADHD child’s emotional experiences. It can shed light on the connections between his/her inner world and symptoms, such as attention deficits, hyperactivity, and impulsivity. On the other hand, it cannot establish causality in the individual or general case. If we recall that the diagnosis is based on a list of symptoms, not of etiology, we realize that this limitation applies to any scientific perspective on ADHD. Psychoanalytic treatment is one of several approaches to understanding ADHD and helping the child cope with it. This is achieved by the psychoanalytic method, a hermeneutic approach with which the analyst interprets the child’s behaviours and communications as they emerge in the session. The implications of such an approach are discussed.​​​​

If we adhere to the call of papers for this issue of the journal, to apply a critical yet informed perspective to the ADHD concept, it would be easy to link it with the critique against the overuse of the diagnosis and stimulants (Batstra & Frances, 2012; Frances & Batstra, 2013). Such critique forms part of another and broader one, namely, against any reductionistic perspective on mental illness. Others have suggested a “third way”. For example, the field of epigenetics highlights and recognizes the influence of experience on a neural level without disqualifying the predictive capacity of genes. Frances (2016) speaks of a “civil war” between the two major approaches of explaining mental disorders; the biological and the psychosocial. The former is “mindless” in assuming that “genes are destiny and that there is a pill for every problem” (p. 58). The latter is “brainless” in suggesting that “mental health problems all arise from unpleasant experience” (p. 58). Frances points out that Freud, who founded psychoanalysis, the most influential school in the latter tradition, often linked his psychological theories with the insights of the neuroscientific knowledge of his era. For example, in his speculations on the death instinct and human aggression, he hoped that biology, “truly a land of unlimited possibilities”, might one day “give us the most surprising information and we cannot guess what answers it will return in a few dozen years to the questions we have put to it” (Freud, 1920​​​​). Indeed, he considered psychoanalysis as an “intermediary between biology and psychology” (Freud, 1913, p. 182). Frances rightly salutes Freud for his commitment to combining biological and psychological perspectives.

The question in this paper is what a psychoanalytic perspective might add to the understanding of ADHD in children. It takes Frances’ warning seriously and does *not* juxtapose a psychological perspective against biological or sociological theories. Rather, it will clarify that the resolve of combining biological, sociological, and psychological perspectives to understand ADHD, though this be important and laudable, is restricted by which instrument of research one is using. Every instrument has been developed for a specific field of knowledge. Thus, instruments from the biological sciences can investigate how ADHD is associated with, for example, variations in ​​​​neurophysiology, neuroanatomy, neurochemistry, diets, and blood chemistry—and nothing more. Sociological theorizing and field studies can reveal important factors on macro- and micro-developments on the social field—and nothing more. Findings from the “psychoanalytic laboratory”, that is, experiences gathered during individual treatments, can help us understand *how the ADHD child experiences having these symptoms and how they connect with his affective world*—and nothing more. In order to present how this “lab work” is being conducted, qualitative data of a boy with ADHD and my interpretations of them will be briefly presented. The aim is to focus on one point in this issue’s appeal to discuss ADHD from “a broad range of non-neurobiological perspectives and contexts”, namely, “the child’s inner drama, symbols and imaginations”. It is hoped that when various sciences, including neuroscience, are combined, a more comprehensive picture of ADHD will emerge. This adheres to Zabarenko’s (2011) suggestion of fielding a team of psychoanalysis, neuroscience, and cognitive psychology.

When I trained in adult psychiatry and psychoanalysis at the turn of the 1980s, the term MBD (minimal brain dysfunction) was in circulation. When I began child analytic training 1990, terms like ADHD and neuropsychiatry had begun to emerge. I did wonder about the name of this new science, neuropsychiatry. Did it imply that one could now link, or would one day be able to link, certain symptoms to a specific cerebral genesis? Without dismissing the findings of neuroscience, I knew that similar claims from earlier days regarding other mental diseases had proven to be simplistic. There had been, and still is, a struggle between those who thought of psychiatric diseases as uniform entities with one single etiology, and those who thought in terms of symptom spectra with multifactorial etiologies. This tension could be observed in discussions about schizophrenia, depression, and neurosis. It was therefore surprising to see the term “neuropsychiatric disorder” emerge, connoting that the symptoms were caused by biological factors, still unclear but to be established in the future.

## Martin, a clinical example

A diseased brain might scare any psychotherapist, especially a freshman taking on children in treatment. When I met my first child patient, five-year-old Martin, his parents had informed me that he had been diagnosed—after thorough investigations at a child psychiatric clinic—with DAMP (deficits in attention, motor control and perception; Gillberg, 2003). In the 1990s, this diagnosis was embraced by the Scandinavian public and Gillberg was its undisputed authority. He emphasized neurobiological factors as causing DAMP, whereas psychosocial risk factors “do not appear to account for the condition as such, but rather for many of the comorbid psychiatric, behavioural, and emotional problems” (p. 907). Later, others would claim that the term DAMP did not carry any explicit etiological assumptions but rather adopted a contemporary “DSM orientated, phenomenological approach to diagnosis” (Sonuga-Barke, 2003, p. 115). Such perspectives and other critical expert papers (Rutter, Taylor, & Hersov, 1994; Rydelius, 2000), appeared years after I first met with Martin. Here, it will suffice to state that the symptoms of DAMP and ADHD are rather similar, the main difference being that DAMP includes dysfunctions in motor control and perception.

As we shall see, the classic symptoms of ADHD, such as problems with attention, impulsivity and hyperactivity, were present in Martin’s case. Note that the purpose of the vignette is not to provide a full description of family background, previous psychiatric examinations, or psychotherapeutic technique. It is rather to illustrate various perspectives on the etiology of ADHD and the epistemology of psychoanalysis in relation to other disciplines.

The parents sought help because Martin was in deep trouble at preschool. He attacked the other children physically and verbally, and the staff was worried. “They just see him as a problem, and the kids pick on him. But they are also frightened of him”, said the concerned mother. An earlier attempt at having Martin with a childminder had worked alright as long as he was her only child. But when a new child arrived, he became aggressive. I also learnt about violent rows at home, about which we soon will learn more.

I set up an appointment with Martin, who was to be followed by his mother to the interview. My attitude when meeting with him was a mixture of curiosity based on psychoanalytic experience, and naivety due to a scant knowledge of the neuropsychiatry literature—a lack that I repaired during this and other therapies with other boys with ADHD. I simply set out to try to understand how his behaviour, thoughts, and emotions were related on conscious and unconscious levels. Here are notes from the very first session, which will serve as an entry into a discussion on what psychoanalysis can, and cannot, contribute with to our understanding of ADHD, and what characterizes its hermeneutic method.

## First encounter with Martin

He enters cheerfully with mother, who sits down in the corner of the room. I have placed some soft animals and other play material on a little table. He immediately becomes interested and picks up a fox, a panda bear, and a penguin.

Martin: The penguin is called Pengy. He’s you.

He builds a corral with a bear and a panda, and the other one with Pengy. He is friendly, cheerful, and inventive.

Martin: The fox eats meat. But he can’t get enough of it. No, he’s eating monsters. They don’t mind being eaten!

Later he adds that the fox eats the other animals while they are asleep. The bear and the panda are five years old. Panda has bad dreams, ghosts are chasing him, and he needs protection.

Analyst: You said Panda needs to be protected. Who is protecting him?

M: The fox! He eats the ghosts and then he’s not hungry anymore. Once everything was gone from Panda’s house; the stove, all the food, the fridge, the cutlery. The fox ate it all.

A: So the fox is protecting Panda but he took all his food.

M: Panda was alone, he had no friends but the fox. Yeah, maybe he’s freezing, ‘cause he has no stove anymore. He told Pengy, “Help me, give me breakfast, I’ve got no food at home!”

Suddenly, Pengy eats all the fox cubs. Martin rips up Pengy’s tummy. The atmosphere has changed. He is excited, moves about in agitation and seems on the verge of becoming violent, perhaps also against me.

## The epistemology of the psychoanalytic method—some clarifications

First some words about how to introduce the method to Martin. One might surmise that a child ignorant of psychotherapy would need explanations. However, for a child as young as Martin I think such a procedure is redundant and even incomprehensible. In contrast, the clinician needs to explain to the parents how s/he intends to work with the child. Often, the parents will find their own way of introducing therapy to the child. If asking for advice I respond, “Tell your child you have been talking to a doctor about the rows at preschool. He likes to talk with children and he’d like to see you”. If the therapist thus has clarified the setting to the parents and they have talked briefly to the child, it is surprising how easily and unabashedly s/he starts laying the cards on the table and outline his​​​​ internal conflicts. If the child is more hesitant, the clinician needs to patiently wait until s/he can insert a little comment about a drawing or a game to raise the child’s interest in therapeutic reflections.

One might conclude that Martin is just a cheerful and unabashed boy who plays imaginatively with somebody he never met before. If I admit that I became worried and concerned during our interchange, this might simply be based on my bias via reports by the parents whom I had met a week earlier. They had told me that Martin is their first child, whereas the mother has a much older child from a previous marriage. This child has no problems, they report. Martin was breastfed for six months. At first, the parents report that problems began when he was one year old and bit his mother violently in her leg. Then they add that up to 3–4 months of age, he had been a very calm boy. Then he changed and they began to experience him as demanding and aggressive. As reported above, his aggression continued at the childminder and later, at preschool.

Today at home, one of the parents can have a cosy time with him at bedtime. Then he starts a quarrel. “It’s as if his anger creeps out of his body”, says the mother. He gets accusatory, “but at the same time he seems to feel pushed up against the wall, as if he were threatened”. He becomes enraged, runs around and a row ensues. He demands the parents to apologize for having started the row, which they refuse, “because that wouldn’t be right”.

Based on this report, I could conclude that Martin’s behaviour in the consulting room was similar to his habits at home; he was preoccupied with biting, seemed increasingly hyperactive, and had a faulty impulse control. A psychoanalytic perspective also aims to go beyond describing behaviours; to intuit his inner world and suggest how it relates to external behaviours and relationships. In discussing the difference between psychiatry and psychoanalysis, Sigmund Freud (1916–1917) suggested that psychiatry “sets about describing the mental disorders it observes and collecting them into clinical entities”. It can only claim to be setting up “descriptive hypotheses” about mental disorders, which “are only accessible to therapeutic influence when they can be recognized as subsidiary effects of what is otherwise an organic illness. This is the gap which psychoanalysis seeks to fill. *It tries to give psychiatry its missing psychological foundation*” (pp. 20–21, italics added). The gap is filled via a prescribed method called psychoanalytic treatment. The analyst takes on this task without any speculations on the etiology of the disorder. Heredity and experiences are linked in a “complemental series” (Freud, 1916–1917, p. 347) and the analyst cannot establish an individual’s place on this sliding scale.

To “fill the gap”, the analyst *interprets* “the patient’s unconscious mental life as it is expressed in the patient’s speech, thoughts, affects, fantasies, and behaviors” (Tuckett & Levinson, 2010, chapter on “Interpretation”). An interpretation is always offered “in the context of the transference/countertransference relationship and therefore carries both conscious and unconscious, transference-laden meanings for the patient” (2010, chapter on “Interpretation"​​​​). The term “transference” implies that the patient is prone to develop a “coloured” view of the therapist. S/he will tend to attribute to the clinician feelings, inclinations, and character traits that seem alien, fantastic, or childish. The term “countertransference” refers to the therapist’s various emotions that are aroused in encounters with the patient.

No matter where we place psychoanalysis along the line between biology and psychology, its *instrument of research* is based on the principles of hermeneutics—not those of natural science. The clinician seeks to bring out “the latent meaning in what the subject says and does” (Laplanche & Pontalis, 1973, p. 227). Today, the act of interpreting is increasingly viewed from an “anti-authoritarian” (Kernberg, 1997) position, as a joint project undertaken by analyst and patient. Inevitably, an interpretation cannot be merely *objective* in the sense that this term is used in the natural sciences. It also reflects the analyst’s “creationism” or *subjectivity* (Ahumada, 1994). For further discussions on psychoanalysis and hermeneutics, see Bouchard (1995), Franke (1998), Friedman (2000), and Laplanche (1992).

The act of interpretation can also be seen as a process, in which the analyst perceives the patient’s communications and makes assumptions that are based on an inductive mode of thought. As s/he suggests them to the patient, both participants can utilise the responses to confirm or disconfirm these guesses. This is done through alternate processes of deduction and abduction and renewed efforts at induction, in a continuous epistemological process as described by the philosopher C.S. Peirce (Kloesel & Houser, 1992, 1998; Muller & Brent, 2000; Rennie, 2012; Salomonsson, 2014). Such a process cannot yield one explanatory model of a certain disorder that would be valid for all patients with that disorder. This is because there exists a “tension between idiographic and nomothetic approaches toward science, with [hermeneutics being] interested mainly in understanding individuals and their particular, idiosyncratic history, beliefs, and behaviors, [and positivism] focused on discovering lawful regularities across individuals” (Luyten, Blatt, & Corveleyn, 2006, p. 580). Similarly, Laplanche (1992) speaks of a tension between a “realistic standpoint”, which seeks to explain the patient’s disorder by recovering his real history—and a “creative hermeneutic” position, which acknowledges that our interpretations are essentially constructions. Applied to Martin, the first position would imply “Certain events occurred in his life and therefore he got ADHD”. The second would entail that “I, as subject, interpret that his ADHD might be linked to experiences that he relates to me”.

Let us bring these issues to the role of psychoanalysis in understanding ADHD. As for the validity and reliability of the ADHD concept, in my view psychoanalysis has nothing to contribute. If psychiatric researchers have found consistent validity of certain symptoms and decided to subsume them under the diagnosis of “ADHD”, their conclusions lie beyond the scope of psychoanalytic investigation. If psychiatrists—as some but far from all do—claim that it is caused by hereditary or traumatic brain dysfunction, a therapist cannot have any view pro or contra *qua* psychoanalyst. On the other hand, these psychiatrists cannot use such a claim to disqualify psychotherapy. What an analyst *can and should* do is to investigate findings in the consulting-room, and draw conclusions from them. For example, the links between ADHD symptoms and emotional experiences are a vital topic where child analysts have much to say. I have found that hyperactivity and impulsivity are often unleashed when the child is overwhelmed by unmanageable emotions. Dysfunctional affect regulation and ADHD symptoms thus seem connected and we need to find out when and why this occurs. What about the external reliability of these findings? As Friedman (2000) puts it, when we practice hermeneutics in psychoanalysis or in other disciplines, it is insufficient to say that we aim to understand the individual text or client. We also engage in an inductive process, in which we make abstractions. As to whether my experiences with Martin might apply to other children, I take a pragmatic view; it is important to investigate children with ADHD in psychoanalysis to learn more about the minds of other children with similar problems.

Having discussed the gap between positivism and hermeneutics, what is then the difference between psychoanalysis and qualitative hermeneutic research methods? Among the latter, many are based on Freud’s use of the psychoanalytic interview (Kvale, 1999; Rennie, 2012). If we follow Rennie’s classification, such research looks both for *experiential* and *discursive* elements in interviews with respondents. Similarly, in the analytic session, I interpret both Martin’s experiences and how certain discursive elements, such as “Blah blah, you’re an idiot” emerge in various emotional situations. Psychoanalytic investigations use single-case methodology, as is also the case with qualitative research methods such as case studies, narrative approaches and interpretative phenomenological analysis (IPA; Smith, Flowers, & Osborn, 1997).

Specific to psychoanalysis is that its practitioners rely on a corpus of theories, which, though encompassing many divergences, contains some fundamental assumptions. The most important is the idea of an Unconscious, which may clash with conscious wishes and cause the patient suffering. If clinical progress is to be achieved, these forces need to be interpreted in an analytic setting. This procedure resembles that of Gadamer’s (1975/1989) understanding of how to interpret a text. He encourages us to be sensitive to its alterity, that is, we must be aware of the chasm separating us from it. For this sensitivity to come about, we must be aware of our bias, or else we become victims of the “tyranny of hidden prejudices” (p. 282). In other words we need to be aware, as far as possible, of our “fore-meanings” or preconceptions, “so that the text [and the patient] can present itself in all its otherness” (Gadamer, 1975/1989, p. 282)​​​​. I recognized Martin’s alterity *and* acknowledged the tradition that supported my interpretations; psychoanalytic theory and practice.

After this digression into epistemology, we now need to address a question on child therapy. If Freud was standing between biology and psychology, and between positivism and hermeneutics, his research method was decidedly hermeneutical. Importantly, though, he applied it to *adult* patients. The idea that one could similarly analyse a *child* patient’s actions was initially seen with hesitant interest (Freud, 1909). Soon some colleagues, mainly Melanie Klein (1932) and Anna Freud (Sandler, Kennedy, & Tyson, 1990) claimed that a child’s play in the consulting room was comparable to an adult’s verbal communications. It contained latent meanings about the child’s wishes, fears, and fantasies, and s/he was amenable to interpretations that would arouse attention, defences and, ultimately, growth. These views form the basis of today’s psychodynamic child therapy and psychoanalysis, and it was in this tradition that I worked with Martin; to let him play and talk while I observed, reflected, and asked questions. At this very first interview, it was way too early to suggest any interpretations to him.

## Ethical considerations

Presentations of clinical material from psychoanalyses or psychotherapies pose challenges regarding confidentiality, consent, and the concealment of identifiable details. The problem has been discussed repeatedly in the literature. For example, Aron (2000) has pointed out that psychoanalytic writers need not only obtain the patient’s consent for publication, a duty that is incumbent on all scientific writers. Analysts must also investigate what the consent implies to the patient on an unconscious level. In a follow-up article, Aron (2016) ascertained that due to developments in modern society, case material is more accessible today. Should we then altogether stop publishing case material? In response, Aron suggests an individualized approach. Applying it to my publications of child treatments, I follow these principles: (1) to obtain consent from the parents, (2) to conceal or change any detail that can facilitate recognition, (3) to avoid presenting lengthy case studies since they increase the risk of breaching the confidentiality, and (4) to wait some years after treatment has ended before I write up and submit the paper. In Martin’s case, it ended many years ago.

## Interpretations of Martin’s play

Now that we have become familiar with the psychoanalytic “looking-glass” and what we purport to see—and not see—with it, let us return to the session. In the play scene, I take the animal’s behaviours to indicate various aspects of Martin’s personality and how he expects other people to act and think. According to one formula, he seems to think that unless you eat somebody he will eat you. Yet, if you are devoured you will assure the predator that this is just fine. If you are lonely and hungry, you can ask for help from someone you trust—but he will soon prove to be a predator as well. Thus, beneath Martin’s play I discern a gruesome and terrifying internal world dominated by loneliness, cruelty, fraud, and hypocrisy.

Martin was in analysis for four years, four sessions a week. His farewell gift was a glass sculpture; a rock with an eagle’s head inside. After many sessions during which he tried to bite me, I concluded that the eagle indicated how he was still dominated by a predatory “internal object”. This term can be compared to bricks with which the child builds up his inner world. He does this by introjecting experiences that are coloured by hereditary factors, earlier physical and emotional experiences, and projections. A child may experience an internal object as “something bad inside me that tells me to do silly things”. It may also exist in a benevolent and loving form as when a child proudly asserts, “I like me”. In the latter case, it is as if he is housing an inhabitant who is fond of him and helps him maintain a healthy self-esteem. The theoretical underpinnings for the concept of internal object will be expanded further down.

Martin’s efforts at handling the bad eagle object were sometimes successful, sometimes not. My clinical approach was to make him more conversant with it through containment (Bion, 1962a), that is, by adopting a benevolent, attentive, and reflective stance with him. I also interpreted how his feelings and actions, especially the ones in the sessions, were inter-connected. The clinical challenge was that he tended to regard my words as missiles that he must ward off by physical attacks. Sometimes, the interventions were also nurtured by my feelings of humiliation and anger when he bit, scolded, and spat at me. Sessions thus vacillated between violence and calm, rage and love, hope and despair.

One day in the middle of the analysis, he arrives early while I am still with another patient. He rushes in and out of the consulting-room and destroys some object in the waiting-room. During the ensuing session, we address his bad conscience and how it gets bigger the more he is mischievous. I ask him why he also confesses to tricks he did *not* do. To answer, he lies down, puts the leg of a stool to his chest: “Let me die, it’s right, let me die!” I am moved by this display of suicidal despair. However, I am also uncertain about its sincerity. He then plays with a wolf, “That’s me when I’m bad” and an owl, “That’s you ‘cause you know many things”. The wolf is angry with the owl and attacks. The owl gets mad, while the wolf says he wants to die and packs himself up in clay. In the end the owl tries to save his life.

In my interpretation, the wolf represents bad aspects of Martin. It cannot contain anger but lets it out in the shape of a ferocious attack against a wise owl. This transforms the owl into an avenger and the mad wolf turns into a suicidal candidate. Once again, the owl changes, now into a lifesaver. In Martin’s world, life is about survival. One is constantly threatened of being cheated and devoured, and the only weapon is cheat and counter-attack. Help can turn into coercion, friendliness into submission, bonds into bondage.

Working with Martin and other boys with ADHD set me thinking in three main areas: how to (1) define the place of psychotherapy, (2) understand their internal world in psychoanalytic terms and (3) develop an adequate therapeutic technique. This resulted in a series of papers (Salomonsson, 1998, 2004, 2006, 2011).

## ADHD and psychodynamic therapy

I will begin this section with describing the countertransference. I have already indicated that when Martin was in a bad mood and attacked me, I felt denigrated and helpless. I also felt pity and sympathy for his struggle to grasp his unrest. Another countertransference aspect was my impressions of his mother; I felt she was a bit mute and rejecting. She and her husband wanted me to help Martin, but she also showed some restraint and distance that was hard to comprehend. In contrast, her husband was unreserved in seeking help for the boy.

Having thus voiced my countertransference vis-à-vis the mother, I immediately open up for critique. Psychiatrists might claim: “ADHD is not a psychogenic disorder but the consequence of a cerebral dysfunction. Don’t tell us it’s the mother’s fault”. The parents could accuse me of blaming them for the child’s disorder. They could, rightfully, state that they did everything for their child and that I do not grasp the hardships in taking care of such a boy. Indeed, all parents of children with ADHD have gone through lengthy periods, which they experienced as excruciating, worrying, humiliating, and guilt-ridden. One could therefore speculate that the mother was sceptical about me, another know-it-all professional of the kind she had met so many times with Martin, and whom she felt looked at the family with un-empathic eyes.

Let me therefore be crystal clear: I do *not* know the cause of Martin’s ADHD, and I certainly do not claim that my impression of his mother has any causal validity. The diagnosis summarizes a series of symptoms, the cause of which nobody knows, generally or individually. According to Rafalovich (2001), the weakness of any explanatory system of ADHD is revealed by those critics who are “telling us that they are the ones with the answer, the ones who are honing in on the truth. Meanwhile, another explanatory system waits in the wings” (p. 414). An important reason, in my mind, to this repeated error is that one often forgets that a professional can only make statements issuing from *his/her* toolbox. The psychoanalytic toolbox contains many instruments, one of which is to note and reflect on the countertransference toward the child and his parents. In terms of hermeneutics, it belongs to prejudices, which the therapist needs to clarify to himself/herself to sharpen the interpretations.

I can thus *speculate* on the links between my impressions of the mother and Martin’s violent internal world—but I do not claim, “She is the explanation”. Rather I ask, “Might there be an association between baby Martin’s biting his mother and his present behaviour? Why did he bite her? What did he feel? What about her feelings? She experienced his biting as an aggressive act but from his infant perspective, was it an effort at gaining access to her? If so, why did he use such a violent method? What roles did the father play? How did he react? How did the spouses relate to these events? What about Martin’s temperament? Why did it change dramatically at 3–4 months of age?” My assumptions become more well-founded if I base them on experiences with Martin in the consulting-room, which is *my* investigatory lab. There, I can make connections when, for example, we talk about something that makes him sad or embarrassed and he responds with anger and biting. In other words, I try to connect his affects with the ADHD symptoms and help him see the links. This is a task for which the psychoanalytic toolbox is specifically devised.

Were I a neuroscientist who did an fMRI​​​​ showing deviations in Martin’s prefrontal cortex (Barkley, 2006), I could not claim that this caused his symptoms. I would have to content myself with musing on the associations between these deviations and his lack of impulse control. Were I a developmental psychologist, I could bring out studies showing associations between hyperkinetic disorders and externalizing behaviours and maternal pre- and post-natal depression (Chronis et al., 2007; Hay, Pawlby, Angold, Harold, & Sharp, 2003) and anxiety (O’Connor, Heron, Golding, Beveridge, & Glover, 2002; Van Den Bergh & Marcoen, 2004), family adversity (Johnston & Mash, 2001), parenting (Johnston & Jassy, 2007) and non-optimal mother–infant interaction (Becker, Holtmann, Laucht, & Schmidt, 2004; Morrell & Murray, 2003; Olson, Bates, Sandy, & Schilling, 2002; Smeekens, Riksen-Walraven, & Van Bakel, 2007). But once again, I could not assert that they explained why *Martin*, specifically, was so angry and violent.

To repeat, the psychoanalytic method cannot establish any *general* causality behind symptoms. It cannot reject or confirm, for example, that “evidence points to neurological and genetic factors as the greatest contributors to this disorder” (Barkley, 2006, p. 219), or that “ADHD cannot and does not arise from purely social factors” (p. 220). Anyone who purports to know the etiology of ADHD should recall Freud’s (Freud, 1895) etiological equation: we need to differentiate between a disorder’s precipitating causes, the necessary preconditions, the specific cause and, finally, “the concurrent causes, which are not necessarily present every time, and which cannot produce the effect by themselves alone” (p. 135).

Psychological factors like early attachment patterns or family environmental factors do associate with conduct problems (Erikson, Sroufe, & Egeland, 1985; Lyons-Ruth, 1996​​​​; Fearon & Belsky, 2011). Conduct problems and ADHD have a high comorbidity (Jensen, Martin, & Cantwell, 1997; Spencer, Biederman, & Mick, 2007). Such psychological factors are thus *concurrent* causes. When Barkley (2006) writes that “social factors do not create ADHD or contribute through some social mechanism to causing this disorder” (p. 220), I agree with the first statement and disagree with the second. When we analyse the unconscious meanings of a symptom, they might include the child’s *experiences* of a parent. If so, these factors are concurrent causes. This does not imply that all such parents have ADHD children, or that such children experience their parents this way or that the parents are such in “reality”.

​​​​Barkley also says that genetic studies indicate that "shared" (p. 172), that is, general environmental factors account only for a small part of individual differences in ADHD, while a greater part of the variance is due to “nonshared”, that is, personal, non-genetic factors. He recommends us to focus on “those biological, interactional and social experiences that are specific and unique to the individual” (p. 172). The psychoanalytic method focuses precisely on the individual’s interactional and social experiences and, as such, it might establish connections with his/her present state.

As regards the results of psychoanalytic therapy with ADHD children, few systematic outcome studies have been done. ​​​​ The NIMH Collaborative Multisite Multimodal Treatment Study of Children with Attention-Deficit/Hyperactivity Disorder, also known as the MTA study, (Arnold et al., 1997; Swanson et al., 2008) did not include it in the treatment arsenal, and neither the reviews of psychosocial treatments by Eyberg, Nelson and Boggs ( 2008​​​​) nor by Pelham and Fabiano (2008) comprised it. I only know of one systematic study of psychoanalytic therapy (Laezer, 2015). In this Frankfurt study, which was a controlled trial with a naturalistic observational design, 43 children received two years of psychotherapy without medication. 30 children, of whom 60% also were on stimulant medication, received behaviour therapy of shorter duration. The results showed that various symptoms were reduced in both groups but without any between-group differences. The reason for excluding methylphenidate in the analytic group seems to be the authors’ declared negative view of medication. As a result, the results of therapy modes became blurred with those of medication. Possibly, if one had allowed therapy children in need of medication to also get it, there might have appeared between-group effects. To conclude, we need naturalistic, randomized studies of the efficacy of psychoanalytic therapy for children with ADHD—where decisions on medication are not mixed up with randomization to the therapy modes compared.

My personal views on the results of psychotherapy have changed somewhat over the years. In 2004 (Salomonsson, 2004) I concluded that “these children benefit from a well-conducted psychoanalytic treatment. They often quickly become interested in a psychoanalytic discourse, because they want to express and get a hold on their experiences.” (p. 8). Later (Salomonsson, 2011), I wrote that these therapies might “complement standard treatments by helping the child to grow emotionally, and … increase our understanding of the internal world of these children”. Whereas other therapies aim at “regulating and correcting behavior, sometimes also emotions, psychoanalysis aims … to help the child learn about his inner world within the therapeutic relationship. The varieties of this relationship may also help him understand his behavior with classmates and family members” (p. 89).

Today, I am more cautious as to the results. I base this conclusion on analyses that showed non-optimal results on hyperactivity. On the other hand these children, all boys, became less anxious, more calm and more adept in speaking about their feelings rather than acting them out. Half of them were on medication prescribed by child psychiatrists and the consumption was unaffected by therapy. All continued to need special pedagogy at school, and in general the teachers became more confident that they would manage school well. Some indicated, at follow-up interviews several years post-treatment, certain remaining issues with attention whereas impulsivity was no longer a problem. Some were seriously committed in love relationships.

## Psychoanalytic conceptualizations of ADHD

Analysts have hesitated to publish papers on ADHD treatments. Perhaps many, following the zeitgeist, regard it as a neurological disease that should not be treated with psychodynamic therapy. Child psychiatrists in Sweden and elsewhere only rarely suggest it for ADHD children. This restricts the possibilities for analysts to become acquainted with the disorder and to understand how these children’s internal worlds are involved in the symptoms. Nevertheless, some have embarked on analysing ADHD children and contributed to theory development. Rainwater (2007) suggested that the symptoms represent a *manic defence* (Winnicott, 1935) by which the child seeks to avoid his “emotionally intense inner reality” (p. 74). He quotes Melanie Klein (1935) in that these emotions arise after the child has projected aggression on another person and then fears retaliation. In the next step, the child feels depressed and remorseful due to the projections and seeks to restore the object. This struggle results in hyperactivity, which represents the child’s effort at avoiding “the pain of internal reality” (Rainwater, 2007, p. 80). It is as if the purpose of hyperactivity is to run away from one’s emotional turmoil. Rainwater summarizes that ADHD behaviour may express the child’s defence against “unconscious depressive and perhaps terrifying forces in his inner or outer experience” (p. 82). I believe this model applies fairly well to Martin’s case. The biting animals testify to his oral aggression. The scene where I ask why he confesses to tricks he is innocent of and he answers “Let me die, it’s right” attests to his remorse. Yet, since hyperactivity seems hard to influence by therapy alone one may wonder if it, apart from the psychodynamics that Rainwater suggests, also might represent a neuronal dysfunction.

Leuzinger-Bohleber and Fischmann (2010) in the Frankfurt study of ADHD children also apply an object relations model based on Klein’s and Winnicott’s concepts, though with a slightly different emphasis. They contend that the child harbours negative affects which produce “extreme images of a hateful being” (p. 148) in him. These *aggressive self-images* are then projected onto the other person who, in consequence, is experienced as a *persecutor*. In contrast to Rainwater, they bring out ADHD symptoms as a result of schizo-paranoid anxieties. This term implies that the child sees other people in black and white, as friends or foes—and thus tends to fear them. Martin’s views of the owl is a case in point; it is either wise or raging. In addition it shifts in character, from second to second. The German authors observe various structural levels and types of object relations among ADHD children. Therefore they refrain, like the other quoted analytic authors, from assuming only one etiology in ADHD and from proposing a singular psychoanalytic model.

Although many studies published before the 1990s probably dealt with children who, today, would have been diagnosed with ADHD, Gilmore (2000) was one of the first analysts to use that term. She applies an *ego-psychological perspective* in viewing it as a “disturbance in the synthetic, organizing and integrative function of the ego” (p. 1260), the sum of which constitutes the disorder. To her, ADHD is a “complex mixture of neuropsychiatric and neurotic components … [which is] often improved by medication but in many cases optimally treated with concurrent psychoanalysis” (​​​​Gilmore, 2000, p. 1260). The analyst’s interpretations contribute to treatment efficacy in that they address the ego impairment, for example, cognitive problems and unsuccessful efforts at defending against drive impulses. In addition, the analyst should focus on helping the child acknowledge and regulate his affects. Analysis should be combined with parent counselling, remediation and medication (Gilmore, 2002), but “the underlying disturbance in synthetic and integrative capacity is not treated by medications” (p. 387).

The formulations by another US analyst, Sugarman (2006) are akin to those of Gilmore; ADHD children often have problems with *regulation of affects and narcissism*, as well as self- and object relations. “Their minds have difficulty balancing and maintaining a homeostatic equilibrium between the many mental processes and contents necessary for adequate self-regulation” (p. 237). This theme was also addressed by Carney (2002)​​​​ in an issue of the journal *Psychoanalytic Inquiry* devoted to neuropsychiatric disorders. The author brought out the ADHD child’s problems with *self-regulation*, defined as an “interpersonally developing capacity to modulate states of arousal and to organize behavior in meaningful, predictable ways” (p. 299). Sugarman also emphasizes that whether these children have constitutional regulatory limitations or have suffered trauma—the impact of which the analyst can only speculate about—they “develop unconscious fantasies to account for their functional difficulties” (p. 237). These fantasies, he adds, should be psychoanalysed.

Palombo (2001)​​​​ conceptualizes ADHD in *self-psychological terms*. The child’s interactions with family and friends tend to be negative. This prevents a favourable development of his “selfobject functions” (p. 153). This term refers to “an action or communication by the other person that contributes to the development of the self” (Tuckett & Levinson, 2010, chapter on “selfobject”). ADHD children’s interactions with other persons, such as caregivers, “are so tainted by negative feelings that the specific selfobject functions the child requires are not available. Most affected may be the idealizing function—with its correlated experience of self-soothing and self-regulation” (Palombo, 2001, p. 153). It is as if the child feels that he has messed up every relationship, nobody likes him, and therefore he cannot calm down and soothe himself. This was definitely one aspect of Martin’s situation when the parents sought help. Palombo points out that these children’s brittle self-esteem makes them vulnerable to “disjunctive moments … when the child ceases to feel understood by the therapist” (p. 276). For example, the therapist may not acknowledge a significant event in the child’s life or refuse to respond to questions. This may arouse emotional pain and unleash impulsive behaviour. One problem working with Martin was when I sensed that a significant event had preceded his arrival at my office, as when he entered in a miserable mood and started a fight with me. I seemed to get the blame for something that perhaps had occurred at preschool and now, he projected helplessness and anger at me. Since he was reticent in talking about it, I often chose just to portray his present mood: “You seem to have a hard time today, Martin”.

In Günter’s (2014) comprehensive review of analytic models of ADHD, he brings out *three perspectives*. One is to view symptoms as “defence formations against early traumatic experiences which the infantile ego was unable to process and integrate” (p.46). Secondly, especially boys are often left without “the means of internalizing a stable boundary-setting agency and to regulate themselves by orienting their actions to such a model” (p. 47). In short, they suffer from the absence of a functioning father figure. Günter thus specifically brings out the child’s *representations* of the father and the need to analyse why they are often sadistic and violent. His third perspective views ADHD as “a malfunctioning of thought and affect processing: sense data and affects, that is to say beta-elements, cannot be digested in appropriate ways to form thoughts and so be transformed into alpha-elements​​​​. Instead they shoot directly into impulses and thus into motor restlessness forming the symptoms of impulsivity and hyperactivity” (p. 49). He uses Bion’s (1962a, 1962b) model of how the psychic apparatus is inundated by sensory beta-elements which, through a process called the *alpha-function*, are transformed into symbols or *alpha-elements*. This process presupposes a person "on the other side", such as a parent, a teacher, or a therapist, who is prepared to contain the child’s anxiety-ridden emissions of beta-elements. A similar idea is expressed in other terms by Sugarman (2006) who addresses the child’s “failure to learn to use verbal symbolizations to express emotion, thus leaving imperative action as a prominent mode of avoiding being overwhelmed by unbearable feelings” (p. 238).

In previous articles, I have addressed the ADHD child’s thought processes (Salomonsson, 2004) and his/her hypersensitivity to analytic interventions (Salomonsson, 2006). I link the problems with how to think about his/her affective experiences to the fact that wishes and affects cannot be adequately contained. I have explained this as being due, not only to compromised cognitive functions in general but, from another perspective, to malevolent internal objects as described in the vignette with Martin. In junction with Leuzinger-Bohleber and Fischmann, and perhaps also with Rainwater, I argue that the child easily interprets the analyst’s containment as a punishment for his sadistic attacks rather than as a benevolent effort at helping him. Therefore, contrary to the analyst’s aim, containment can jeopardize the child’s thought processes in that *the transformation of beta- into alpha-elements is blocked*. The result may be that bizarre objects accumulate in the child’s internal world. They may appear in drawings, in words or in actions. Indeed, we recall quite a few bizarre components in the animals introduced by Martin.

In ego-psychological terms, the child’s thoughts become fragmented and his communicative abilities deteriorate. Sometimes, the child projects such objects onto the analyst who suddenly may appear frightening or alien and is therefore attacked. In addition, the child’s memory function is compromised, which prevents him from buffering a frustration by recalling that the analyst was also experienced as a helpful and wise owl, to speak with Martin. As a result of his “temporal myopia” (Barkley, 1998​​​​, p. 247) the child easily panics when he fails to recall such a satisfying object. In treatment, one may see how the child suddenly forgets a recent experience of the analyst as a supportive figure.

This model of ADHD combines *object-relational and ego-psychological perspectives*. In the next section, I focus on a phenomenon discovered in treatments with ADHD children: a bad, un-containing internal object, which is easily awakened in the session and exhorts the child to expel the analyst’s words. This object is affected by the present transference relationship. It also negatively affects the child’s ego-functioning, and here I focus on one aspect not listed among the common ADHD symptoms: the ego’s faltering semiotic capacity, that is, a vacillating ability to use signs for thinking and communicating thoughts and feelings. This may suddenly crumble in an ADHD child, and one can observe how it is continuously influenced by the child’s emotions vis-à-vis the therapist. Accordingly, his ability to receive and muse on analytic interventions may vary from minute to minute, a phenomenon which the clinician must take into account.

## The internal object

Though Freud did not use the term internal object, its origin can be traced to his term “the super-ego” (Freud, 1923). Some years earlier (Freud, 1917) he had spoken of how “one part of the ego sets itself over against the other, judges it critically, and, as it were, takes it as its object” (p. 247). He emphasized that, especially among patients with melancholia and obsessional neurosis, vast areas of this part of the psychic apparatus are unconscious which makes the super-ego function as an invisible and relentless judge. In Kleinian theory, the terms “internal object” or “introject” have become expanded to cover the “‘personal relations’, however primitive and fantastic, we have had with the figures who people our inner worlds” (Riviere, 1955, p. 347). It refers to “an unconscious experience or phantasy of a concrete object physically located internal to the ego (body) which has its own motives and intentions towards the ego and to other objects” (Hinshelwood, 1989, p. 68). It mirrors reality but also contributes, through projection, to how we perceive external objects. To sum up, our object relations are moulded by an “interaction between introjection and projection, between internal and external objects and situations” (Klein, 1946, p. 99).

The character of an internal object is determined by “the attributes of the introjected parent with which the child is predominantly concerned at the moment” (Heimann, 1989, p. 137). Please note that Heimann speaks of the introjected parent, not the real one. In analysis, the child is concerned with how s/he perceives the analyst. When Martin focuses on my “intelligence, skill, manipulation of things—functions belonging to the intellectual and motor sphere of the ego—the introjected object is mainly taken up into the child’s ego” (1989, p. 137​​​​). This is represented by the owl who “knows so many things” and with whom Martin tries to identify. But then, love and hate clash and the owl switches into assaulting the angry wolf Martin. Such constant movements between war and peace give the super-ego an unyielding authority. It is like having a “devil inside” (A. Freud, 1929, p. 33). The internal good object is too weak to function “as a focal point in the ego” (Klein, 1946, p. 101). This is one way of explaining how Martin’s weak semiotic abilities made him receive my interpretations about his loneliness in the waiting-room. He took them as my revenge because he had hit me. In Bionian terms he could not create symboligenic alpha-elements out of my interventions. Due to this semiotic frailty he often feels uncertain whether I mean what I say or, alternately, if my words are harsh exhortations or sly attacks. When Martin is calm and feels well, he reads me on the communicative level that I intend to convey. But when he is agitated or threatened, my words are received as missiles and he must defend himself physically. In addition, his memory function is compromised so he cannot retain the friendly Pengy or the wise owl as good internal objects.

## Conclusions on therapeutic technique

As said, the purpose of the paper and the case vignette is not to provide a review of therapeutic technique. For more extensive comments on this topic, I refer to my previous publications and the papers cited above. The following points are submitted merely to bring out some specificities when working with these children. They stand out as conclusions based on the observations and theories on the child’s semiotic fragility, the brittle internal object, the defensive processes and the ever-present guilt and shame. Taken together, these burdens impose on the therapist a challenge to take special caution and adapt his/her technique. The internal object’s fragility requires him/her to be vigilant as to its present state when s/he, for example, considers interpreting affective content or utilizing a metaphor. Since the child moves rapidly between different semiotic levels and you often are on different levels, the consequence is that what you say, how you say it and how you look and sound combine to a message the child might interpret on another level than you intended.

Countertransference is often intense with ADHD children in therapy. It may emerge as threatened, angry, sad, desperate, humiliated or bewildered feelings. As always, it is wise to reflect on whether they echo the child’s chaos projected into you. Approaching it this way, it can be a valuable informant about the patient’s inner state. Of course, this is impeded if violence occurs. Then the therapist must tell the child s/he will help him prevent it from reappearing since otherwise s/he feels bad afterwards, the internal object is aggravated, guilt feelings increase, and one’s thinking is affected by the assault. The child often understands that a scared analyst cannot do a good job.

I hope to have conveyed how psychoanalytic theory and therapy can contribute with some valuable perspectives on ADHD; not regarding its etiology but its treatment and the child’s experiential world. My recommendation that it should be offered more often than is done today does not in any way conflict with other treatment ingredients that are often necessary, such as medication, pedagogy, or parent training—which should be done by experts other than the therapist.
